# Interleukin-1α expression precedes IL-1β after ischemic brain injury and is localised to areas of focal neuronal loss and penumbral tissues

**DOI:** 10.1186/1742-2094-8-186

**Published:** 2011-12-29

**Authors:** Nadia M Luheshi, Krisztina J Kovács, Gloria Lopez-Castejon, David Brough, Adam Denes

**Affiliations:** 1 Faculty of Life Sciences, University of Manchester, UK; 2 Laboratory of Molecular Neuroendocrinology, Institute of Experimental Medicine, Budapest, Hungary

## Abstract

**Background:**

Cerebral ischemia is a devastating condition in which the outcome is heavily influenced by inflammatory processes, which can augment primary injury caused by reduced blood supply. The cytokines interleukin-1α (IL-1α) and IL-1β are key contributors to ischemic brain injury. However, there is very little evidence that IL-1 expression occurs at the protein level early enough (within hours) to influence brain damage after stroke. In order to determine this we investigated the temporal and spatial profiles of IL-1α and IL-1β expression after cerebral ischemia.

**Findings:**

We report here that in mice, as early as 4 h after reperfusion following ischemia induced by occlusion of the middle cerebral artery, IL-1α, but not IL-1β, is expressed by microglia-like cells in the ischemic hemisphere, which parallels an upregulation of IL-1α mRNA. 24 h after ischemia IL-1α expression is closely associated with areas of focal blood brain barrier breakdown and neuronal death, mostly near the penumbra surrounding the infarct. The sub-cellular distribution of IL-1α in injured areas is not uniform suggesting that it is regulated.

**Conclusions:**

The early expression of IL-1α in areas of focal neuronal injury suggests that it is the major form of IL-1 contributing to inflammation early after cerebral ischemia. This adds to the growing body of evidence that IL-1α is a key mediator of the sterile inflammatory response.

## Findings

Inflammation is recognised as a major contributor to the worsening of acute brain injury [1]. In particular two pro-inflammatory members of the IL-1 family of cytokines, IL-1α and IL-1β, are considered the major effectors of injury, and inhibiting their signalling with the IL-1 receptor antagonist (IL-1Ra) is protective in experimental models of stroke [1], and has shown promise as a treatment in clinical trials [2]. Mice in which both IL-1α and IL-1β have been deleted (IL-1α/β double KO) have markedly reduced damage in response to experimental stroke caused by middle cerebral artery occlusion (MCAo) [3]. However, the relative contribution of each cytokine to the evolution of the infarct is not clear since IL-1Ra inhibits both cytokines. The neuroprotective effects of IL-1Ra are reduced when administration is delayed beyond 3 h [4], suggesting that IL-1 expressed early after the insult is important. IL-1β mRNA is detected within 3-6 h after cerebral ischemia [5,6], although there is very little direct evidence that IL-1β protein is produced, and almost no information is available about IL-1α. In this study we sought to determine the spatial distribution of IL-1α and IL-1β in the mouse brain early (4 h) and late (24 h) after stroke induced by MCAo. Such a study was required since strategies aimed at inhibiting inflammation in the brain will be dictated by the nature of the inflammatory mediators produced.

**Figure 1:**
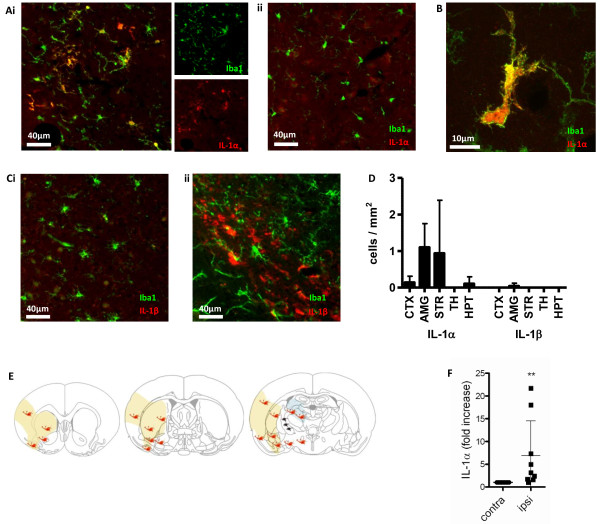
**Early microglial IL-1α expression after MCAo**. Images are of coronal sections from the brains of C57BL6/H mice subjected to 60 min MCAo followed by 4 h reperfusion. Widefield images (A) show IL-1α-expressing (red), Iba1 positive (green) microglia 4 h post-MCAo in the ipsilateral (Ai) but not the contralateral (ii) hemisphere. Confocal image shows the colocalization of IL-1α and Iba-1 stains in an activated microglia in the ipsilateral hemisphere (amygdala is shown) (B). No IL-1β-positive (red)/Iba-1 positive (green) microglia were detected in the ipsilateral amygdala at this time point (Ci, widefield). IL-1β positive, Iba-1 negative cells were detected in the capsula interna (Cii, widefield). IL-1α and β expressing microglia (IL-1/Iba1 positive cells) were counted (D) in the cortex (CTX), amygdala (AMG), striatum (STR), thalamus (TH) and hypothalamus (HPT). n = 3 C57BL6/H mice. The average distribution of IL-1α-positive microglia (orange symbols) and a few IL-1β expressing non-microglial cells (black symbols) are shown at 4 h reperfusion in the ipsilateral hemisphere (E), when histologically little ischemic damage is detected. Yellow shading indicates areas which typically become ischemic by 24 h reperfusion, while ischemic damage is occasionally observed in the thalamus and hippocampus (blue shading) at the same time point. Quantitative real-time PCR demonstrated a significant increase of IL-1α mRNA in tissue homogenates of the ipsilateral hemisphere compared to the contralateral side (F). **P < 0.01, unpaired t test.

We first investigated whether IL-1 expression could be detected early (4 h after reperfusion) after ischemic brain injury, when little neuronal death is present compared to the 24 h reperfusion time. C57BL6/H mice (male, 12-16 weeks) were subjected to 60 min MCAo and 4 h reperfusion following which they were transcardially perfused with saline followed by 4% paraformaldehyde. After cryoprotection brains were cut on a sledge microtome at a thickness of 20 μm and were stored in cryoprotectant solution until use. Immunoflourescence on these sections showed IL-1α expression (AF-400-NA, R & D Systems, 0.4 μg/mL) in microglia in the ipsilateral hemisphere as identified by co-staining for the microglial marker Iba-1 (019-19741, Wako, 1 μg/mL) (Figure 1A, B). At this time no IL-1β expression (AF-401-NA, R & D Systems, 0.4 μg/mL) was observed in these areas, with only a few non-microglial IL-1β-positive cells observed in the capsula interna, away from the core of the infarct, as reported previously [7]. No IL-1 expression was observed in the contralateral hemisphere, confirming that its expression was a result of the injury (Figure 1A). To further confirm early IL-1α expression after MCAo, we performed quantitative real-time PCR on tissue homogenates of the ipsilateral and contralateral hemispheres 4 h after reperfusion. A significant increase (P < 0.01) was observed in IL-1α expression, whereas we were unable to detect IL-1β expression at this time point (Figure 1F).

**Figure 2:**
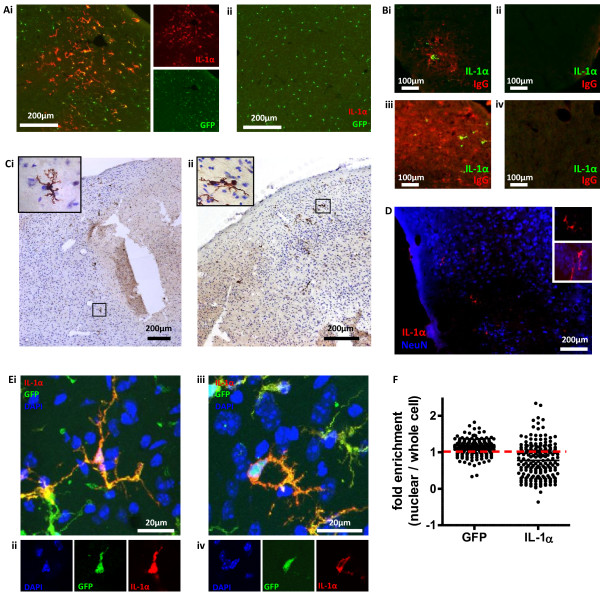
**IL-1α is expressed by microglia localized to focal neuronal and BBB injury 24 h after MCAo**. Images are coronal sections from brains of C57BL6/H and CX3CR1-GFP +/- mice 60 min MCAo and 24 h reperfusion. Widefield images show IL-1α-expressing (red), GFP positive (green) microglia in ipsilateral (Ai), not contralateral (ii) amygdala 24 h after MCAo in a CX3CR1-GFP +/- mouse. IL-1α immunohistochemistry with cresyl violet co-staining localises IL-1α expressing microglia to the peri-infarct zone in thalamus (Bi) and cortex (Bii) of a C57BL6/H mouse. Focal IgG staining (red) co-localized with IL-1α positive microglia (green) in the ipsilateral cortex of a C57BL6/H mouse (Ci). No IgG or IL-1α staining detected in the contralateral cortex (Cii). IL-1α positive microglia detected in larger areas of IgG staining in the ipsilateral (Ciii), but not contralateral (Civ) hemisphere. Co-localization of IL-1α positive microglia (red) with areas of neuronal loss (blue) in a C57BL6/H mouse (D). Occasional IL-1α positive microglia also found in areas where neurons were morphologically intact (D, inset). Confocal images (E) are maximum Z projections (Ei, iii) and confocal slices at the level of the nucleus (Eii, iv) of IL-1α expressing, GFP positive microglia in a CX3CR1-GFP +/- mouse. Cells with (Ei, ii), and without (Eiii, iv) nuclear IL-1α. Nuclear fluorescence intensities for IL-1α and GFP were quantified from confocal images, and the fold enrichment of IL-1α and GFP in microglial nuclei was calculated in comparison to whole cell fluorescence (F). All images are representative of n ≥ 3 mice. Quantification is of n = 4 CX3CR1-GFP +/- mouse brains, with each data point representing an individual cell, n ≥ 30 cells per brain.

Only microglia of CX3CR1-GFP+/- mice (as used previously [7]) express GFP in the brain [8]. CX3CR1-GFP+/- mice were subjected to 60 min MCAo followed by 24 h reperfusion, when the evolution of the infarct is advanced and there is substantial neuronal death. Ramified, GFP-positive microglia-like cells in the ipsilateral hemisphere expressed IL-1α (Figure 2A). IL-1α-positive microglia were present in the cerebral cortex, the piriform cortex, the ventral striatum and the thalamus. Immunohistochemistry for IL-1α, with cresyl violet co-staining, localized IL-1α expressing microglia mainly to the penumbral tissue surrounding the infarct (Figure 2B). IgG cannot cross an intact blood brain barrier (BBB) and its presence in the brain parenchyma indicates BBB disruption and injury [9]. Staining of coronal brain sections from C57BL6/H mice (Figure 2C) and CX3CR1-GFP+/- mice (not shown) 24 h after MCAo for IL-1α and IgG (BA-2000, Vector Labs, biotinylated horse anti-mouse IgG, 2 μg/mL; S-32356, Invitrogen, Alexa 594 conjugated streptavidin, 5 μg/mL) revealed that IL-1α expressing cells co-localized to areas of focal BBB damage, mainly near the penumbral regions of the ipsilateral hemisphere (Figure 2C). The localization of IL-1α expressing cells with injured brain tissue is also shown by the co-localization of IL-1α positive microglia (red) with areas of focal neuronal loss within the compromised tissue (absence of NeuN (MAB377, Millipore, mouse anti-NeuN 5 ug/mL)) (Figure 2D). IL-1α expressing microglia increased overall after 24 h reperfusion compared to the 4 h time point (two-way ANOVA, P < 0.05, not shown) although the increase was not significant when comparing any particular brain regions. At 24 h IL-1β is expressed [7]. Thus the expression of IL-1α in microglia occurs early after an ischemic insult and is localized to areas of injury, mostly near peri-infarct regions.

IL-1α can be actively localized to cell nuclei [10] and in microglia this may represent a mechanism of inhibiting IL-1α release after hypoxic cellular injury [11]. 24 h after 60 min MCAo in CX3CR1-GFP +/- mice, microglia expressing GFP also expressed IL-1α that was localized to the nucleus in some cells (Figure 2Ei, ii), but not in others (Figure 2Eiii, iv). Confocal images (Leica TCS SP5 AOBS confocal microscope) were captured from these sections and were processed and analysed with Image J http://rsb.info.nih.gov/ij. Regions of interest for quantification of mean fluorescence intensities (MFIs) in whole microglia and microglial nuclei were selected using the GFP and DAPI signals respectively. MFIs were quantified for IL-1α and GFP in whole microglia using a maximum Z projection of the confocal image stack. Nuclear IL-1α and GFP MFIs were quantified in a confocal slice at the level of the nucleus. The fold enrichment of IL-1α and GFP in the nucleus were calculated as follows: Fold enrichment = MFI (nucleus)/MFI (whole cell). The fold enrichment data suggest that GFP was uniformly distributed throughout the cell (Figure 2F). However the spread of IL-1α enrichment throughout a cell was much broader suggesting that its localization between the nucleus and cytosol was regulated (Figure 2F). We have previously reported that IL-1α is retained in the nuclei of dead and dying cells [11], and also that microglia die in the infarct following MCAo [12]. Thus it is possible that microglia in an ischemic brain undergoing cell death processes may localize IL-1α to the nucleus to limit its release.

These data show that IL-1α is expressed by microglia at sites of brain injury within a relevant window of time at which blocking the effects of IL-1 are known to be neuroprotective [4]. At these times, IL-1β is not present suggesting that IL-1α is the active IL-1 isoform in mediating the early inflammatory period following ischemic brain injury. This is consistent with recent discoveries highlighting the earlier appearance of IL-1α in sterile inflammatory responses, with a later contribution from IL-1β [13], although functional evidence for a role of microglia-derived IL-1α in brain injury is not provided here. In cerebral ischemia the primary injury to brain cells is caused by the lack of blood supply and so can be considered sterile. Sterile inflammation is known as a major contributor to disease and injury [14], and IL-1α has become recognised as a major mediator of sterile tissue injury [15,16]. Thus the data presented here extend our, and others, previous work showing that IL-1α is a key inflammatory cytokine following tissue injury. This study also extends our previous *in vitro *studies showing that the sub-cellular distribution of IL-1α is regulated under conditions of hypoxia, which may be relevant to the regulation of its release and sterile inflammatory responses [11].

## List of abbreviations

BBB: Blood Brain Barrier; GFP: Green Fluorescent Protein; IL-1: Interleukin-1; IL-1Ra: Interleukin-1 receptor antagonist; MCAo: Middle Cerebral Artery occlusion; MFI: Mean Fluorescence Intensity.

## Competing interests

The authors declare that they have no competing interests.

## Authors' contributions

NML carried out the immune studies and analysis. KJK contributed to the design and execution of the surgical studies. GLC contributed experimentally. DB contributed to design and analysis of the study and wrote the manuscript. AD carried out the surgeries, contributed to the design and analysis of the study and wrote the manuscript. All authors read and approved the final manuscript.
